# Atomically Dispersed Nickel Anchored on a Nitrogen‐Doped Carbon/TiO_2_ Composite for Efficient and Selective Photocatalytic CH_4_ Oxidation to Oxygenates

**DOI:** 10.1002/anie.202215057

**Published:** 2022-12-16

**Authors:** Hui Song, Hengming Huang, Xianguang Meng, Qi Wang, Huilin Hu, Shengyao Wang, Hongwei Zhang, Wipakorn Jewasuwan, Naoki Fukata, Ningdong Feng, Jinhua Ye

**Affiliations:** ^1^ International Center for Materials Nanoarchitectonics (WPI-MANA) National Institute for Materials Science (NIMS) 1-1 Namiki Tsukuba Ibaraki 305-0044 Japan; ^2^ State Key Laboratory of Materials-Oriented Chemical Engineering College of Materials Science and Engineering Nanjing Tech University Nanjing 210009 P. R. China; ^3^ Hebei Provincial Laboratory of Inorganic Nonmetallic Materials College of Materials Science and Engineering North China University of Science and Technology Tangshan 063210 P. R. China; ^4^ Graduate School of Chemical Sciences and Engineering Hokkaido University Sapporo 060-0814 Japan; ^5^ TJU-NIMS International Collaboration Laboratory School of Material Science and Engineering Tianjin University Tianjin 300072 P. R. China; ^6^ College of Science Huazhong Agricultural University Wuhan 430070 P. R. China; ^7^ Biogas Institute of Ministry of Agriculture and Rural Affairs Chengdu 610041 P. R. China; ^8^ State Key Laboratory of Magnetic Resonance and Atomic and Molecular Physics National Center for Magnetic Resonance in Wuhan CAS Key Laboratory of Magnetic Resonance in Biological Systems Wuhan Institute of Physics and Mathematics Innovation Academy for Precision Measurement Science and Technology Chinese Academy of Sciences Wuhan P. R. China

**Keywords:** Oxygenates, Photocatalysis, Selective Methane Oxidation, Single Ni−NC Sites, TiO_2_

## Abstract

Direct photocatalytic oxidation of methane to liquid oxygenated products is a sustainable strategy for methane valorization at room temperature. However, in this reaction, noble metals are generally needed to function as cocatalysts for obtaining adequate activity and selectivity. Here, we report atomically dispersed nickel anchored on a nitrogen‐doped carbon/TiO_2_ composite (Ni−NC/TiO_2_) as a highly active and selective catalyst for photooxidation of CH_4_ to C1 oxygenates with O_2_ as the only oxidant. Ni−NC/TiO_2_ exhibits a yield of C1 oxygenates of 198 μmol for 4 h with a selectivity of 93 %, exceeding that of most reported high‐performance photocatalysts. Experimental and theoretical investigations suggest that the single‐atom Ni−NC sites not only enhance the transfer of photogenerated electrons from TiO_2_ to isolated Ni atoms but also dominantly facilitate the activation of O_2_ to form the key intermediate ⋅OOH radicals, which synergistically lead to a substantial enhancement in both activity and selectivity.

## Graphical abstract

An atomically dispersed Ni−NC/TiO_2_ photocatalyst was designed for direct CH_4_ oxidation with O_2_ to oxygenates. Single‐atom Ni−NC sites not only promote the carrier separation to facilitate CH_4_ conversion, but also enhance the activation of O_2_ to ⋅OOH radicals, thereby leading to a substantial enhancement in activity and selectivity to oxygenates.

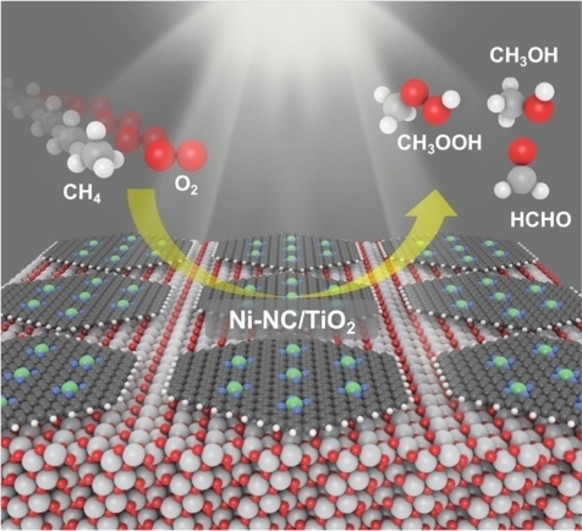

## Introduction

Methane is not only a highly available clean fuel from natural gas, shale gas and biogas, but also a very potent and important greenhouse gas with a warming potential more than 25 times that of CO_2_.[^1^, ^2^, ^3^] The catalytic conversion of methane to higher added‐value chemicals, typically derived from petroleum and coal, is therefore attractive for reducing dependence on crude oil and mitigating global warming. The current industrial methane conversion technology is realized through an indirect route, associated with an energy‐intensive syngas production process and the subsequent methanol or Fisher‐Tropsch synthesis.[^3^, ^4^] Direct conversion of methane to methanol and other oxygenates with molecular oxygen is one of the most ideal approaches to realize methane transformation more efficiently and cleanly.[^5^, ^6^, ^7^, ^8^, ^9^] The key challenge in direct methane conversion is the activation and selective oxidation of methane, because methane is a rather inert molecule and the desired products are more reactive than methane and is susceptible to overoxidation to CO_2_.[^6^, ^7^] To minimize the overoxidation of oxygenates, the methane conversion reaction is generally conducted at relatively low temperatures (<200 °C), along with the utilization of corrosive or expensive oxidants (such as sulfuric acid, N_2_O, H_2_O_2_) to replace O_2_ to activate methane and/or the operation of a stepwise chemical looping process, which makes the process economically uncompetitive.[^5^, ^6^, ^7^, ^10^, ^11^]

Compared with thermocatalytic methane conversion, photocatalytic methane oxidation can proceed at room temperature to achieve appreciable yields of oxygenates and has recently received great interest.[^12^, ^13^, ^14^, ^15^, ^16^, ^17^, ^18^, ^19^, ^20^, ^21^, ^22^, ^23^] Cocatalysts play a vital role in semiconductor‐based photocatalytic methane oxidation reactions, as they can not only promote the separation and transfer of photogenerated charge carriers, but also control the activation of reactants, thereby enhancing surface reaction rates and tuning product selectivity. Among various cocatalysts, noble metals generally exhibit the outstanding performance for photocatalytic methane oxidation.[^15^, ^16^, ^19^, ^20^, ^22^, ^23^, ^24^, ^25^] For example, our previous studies showed that noble metals (Pt, Pd, Au or Ag) decorated ZnO were active and selective for photooxidation of CH_4_ with O_2_ to oxygenates (CH_3_OOH, CH_3_OH and HCHO) and Ag/TiO_2_{001} enabled the selective production of CH_3_OH, while pristine ZnO and TiO_2_ exhibited low activity and selectivity for the production of oxygenates.[^20^, ^23^] Other researchers have also reported a series of good photocatalysts with noble metals as cocatalyst for photocatalytic aerobic oxidation of methane to oxygenates, such as Au−Pd/TiO_2_,^[26]^ Pd/In_2_O_3_,^[16]^ Au/WO_3_,^[25]^ Pt/WO_3_ ^[24]^ and black phosphorous‐supported Au single atoms.^[19]^

Despite the promising results obtained in the above‐mentioned studies, the high‐cost and limited‐reserves of noble metals limits their applications. Therefore, there is a high demand to develop low‐cost and high‐performance alternative cocatalysts to noble metals. Atomically dispersed non‐precious metal atoms anchored on N‐doped carbon (M‐NC) materials, which are generally considered as single atomic site catalysts, have been employed as cocatalysts for efficient photocatalytic reactions such as H_2_ production and CO_2_ reduction,[^27^, ^28^, ^29^] due to the highly exposed active metal sites and efficient transfer of charge carriers. Moreover, the electronic structure of atomic metal sites can be fine‐tuned by changing the coordination environments, rendering M‐NC active and selective for targeted catalytic reactions with favorable reaction kinetics.[^27^, ^30^] In view of such distinctive characteristics, M‐NC potentially have the capability to enhance the activity and selectivity of semiconductor‐based photocatalysts in photooxidation of CH_4_. Nevertheless, to the best of our knowledge, there have been no studies reporting the utilization of M‐NC as cocatalysts for photocatalytic CH_4_ oxidation.

In this work, a single‐atom Ni−NC/TiO_2_ composite is prepared and used as a photocatalyst for direct CH_4_ oxidation with O_2_ to produce liquid oxygenates. We found that owing to the unique structural properties, the atomically dispersed Ni−NC sites not only promote the carrier separation and transfer efficiency, but also enable the controlled activation of O_2_ to ⋅OOH radicals, a key intermediate for the formation of the primary product CH_3_OOH that can be readily transformed into CH_3_OH and HCHO. As a result, a high C1 oxygenates yield of up to 198 μmol with 93 % selectivity is achieved after 4 h of irradiation, superior to most previously reported photocatalysts using noble metals as cocatalysts.

## Results and Discussion

**Figure 1:**
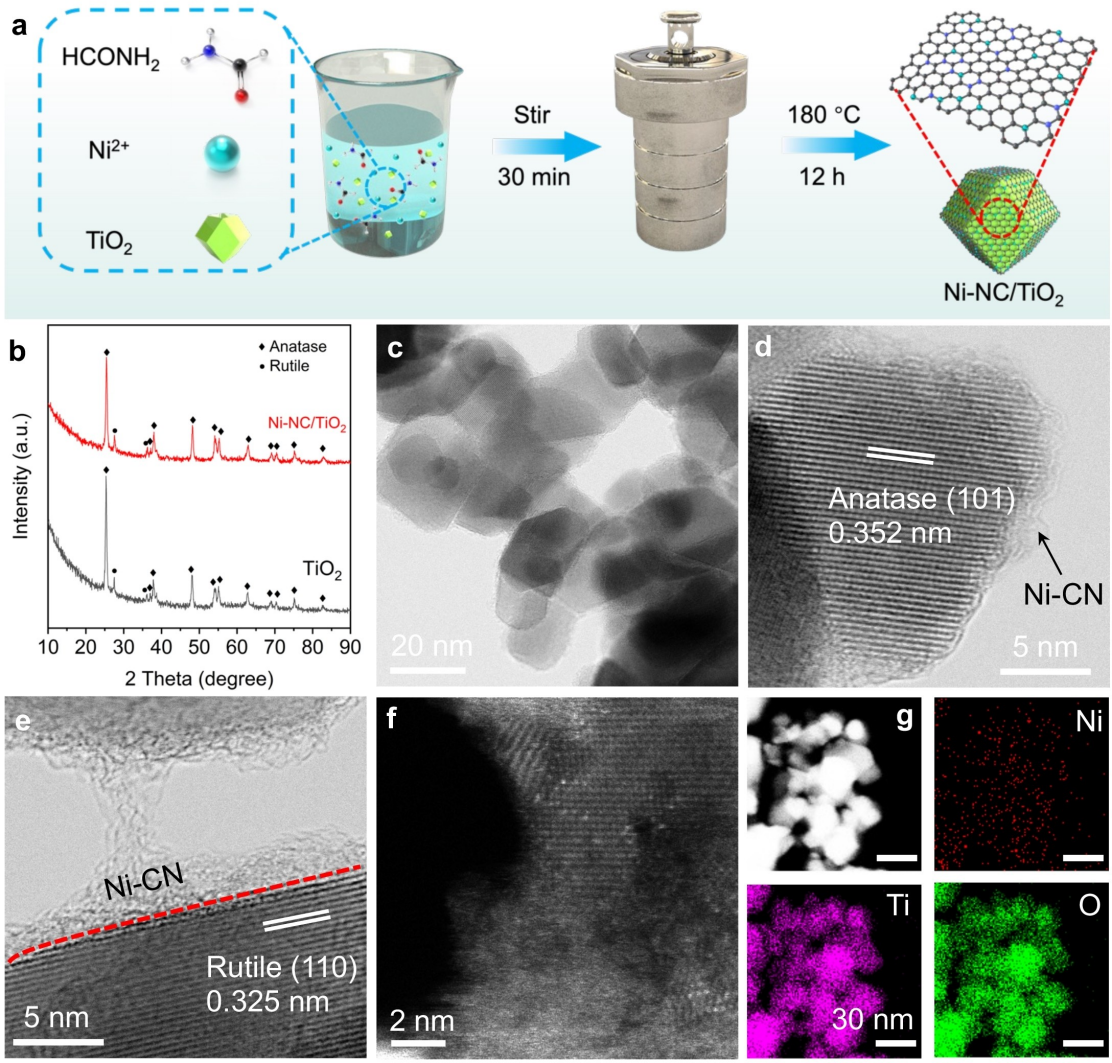
Schematic illustration and morphology characterizations of Ni−NC/TiO_2_. a) Schematic illustration of the synthetic procedure of Ni−NC/TiO_2_. b) XRD patterns of Ni−NC/TiO_2_ and TiO_2_. c) TEM image of Ni−NC/TiO_2_. d) and e) HR‐TEM images of Ni−NC/TiO_2_. f) AC HADDF‐STEM image of Ni−NC/TiO_2_. g) EDX elemental mapping images of Ni−NC/TiO_2_.

Figure 1a illustrates the synthetic process for the preparation of Ni−NC/TiO_2_ via a facile one‐pot solvothermal method.^[31]^ Briefly, TiO_2_ (P25) and Ni precursor (NiCl_2_) were first dispersed in formamide (HCONH_2_). Then, the mixed solution was solvothermally heated at 180 °C for 12 h. During the solvothermal process, formamide can be easily transformed into nitride‐doped carbon (NC) material on the surface of TiO_2_; meanwhile, considering the strong interaction of Ni−N coordination, Ni−N bond was formed in the presence of Ni^2+^. Finally, the resulting sample was washed with diluted acid and water for several times to yield TiO_2_ loaded with NC coordinated Ni catalyst (denoted as Ni−NC/TiO_2_). The color of the material after solvothermal reaction changed from white to black (Figure S1), indicative of loading of Ni and CN on TiO_2_. The Fourier‐transform infrared (FT‐IR) spectra show a new peak at 1386 cm^−1^ on Ni−NC/TiO_2_ (Figure S2), confirming the presence of C−N groups. Inductively coupled plasma optical emission spectrometry shows that the weight amount of Ni in Ni−NC/TiO_2_ is 0.5 wt %. For comparison, TiO_2_ decorated with Ni nanoparticles (NPs) with a loading amount of 0.5 wt % (denoted as Ni NPs/TiO_2_) was prepared via an impregnation method followed by H_2_ reduction at 400 °C for 1 h.

X‐ray diffraction (XRD) patterns (Figure 1b) show that all diffraction peaks are associated with TiO_2_ (anatase and rutile) and no peak of any likely Ni species is observed on Ni−NC/TiO_2_ and Ni NPs/TiO_2_.^[32]^ Transmission electron microscopy (TEM) and high‐resolution TEM images (Figure 1c–e) show that the surface of TiO_2_ is wrapped by a thin amorphous layer in Ni−NC/TiO_2_ and no sign of remarkable Ni NPs is detected, while small Ni NPs with size of 2–3 nm were formed on TiO_2_ surface in Ni NPs/TiO_2_ (Figure S3). Two lattice fringes with interplanar distances of 0.352 and 0.325 nm agree well with the crystal parameters of anatase (101) and rutile (110) planes, respectively, implying that solvothermal treatment did not alter the crystal structure of TiO_2_ (Figure 1d and e). Aberration‐corrected high‐angle annular dark‐field scanning TEM (AC HAADF‐STEM) image shows many isolated bright spots with no observed clusters or subnanoparticles in Ni−NC/TiO_2_ (Figure 1f), which directly validates the formation of atomically dispersed Ni sites. The energy dispersive X‐ray (EDX) spectroscopy elemental mapping analysis (Figure 1g) demonstrates that elemental Ni is uniformly dispersed throughout the entire structure of Ni−NC/TiO_2_.

**Figure 2:**
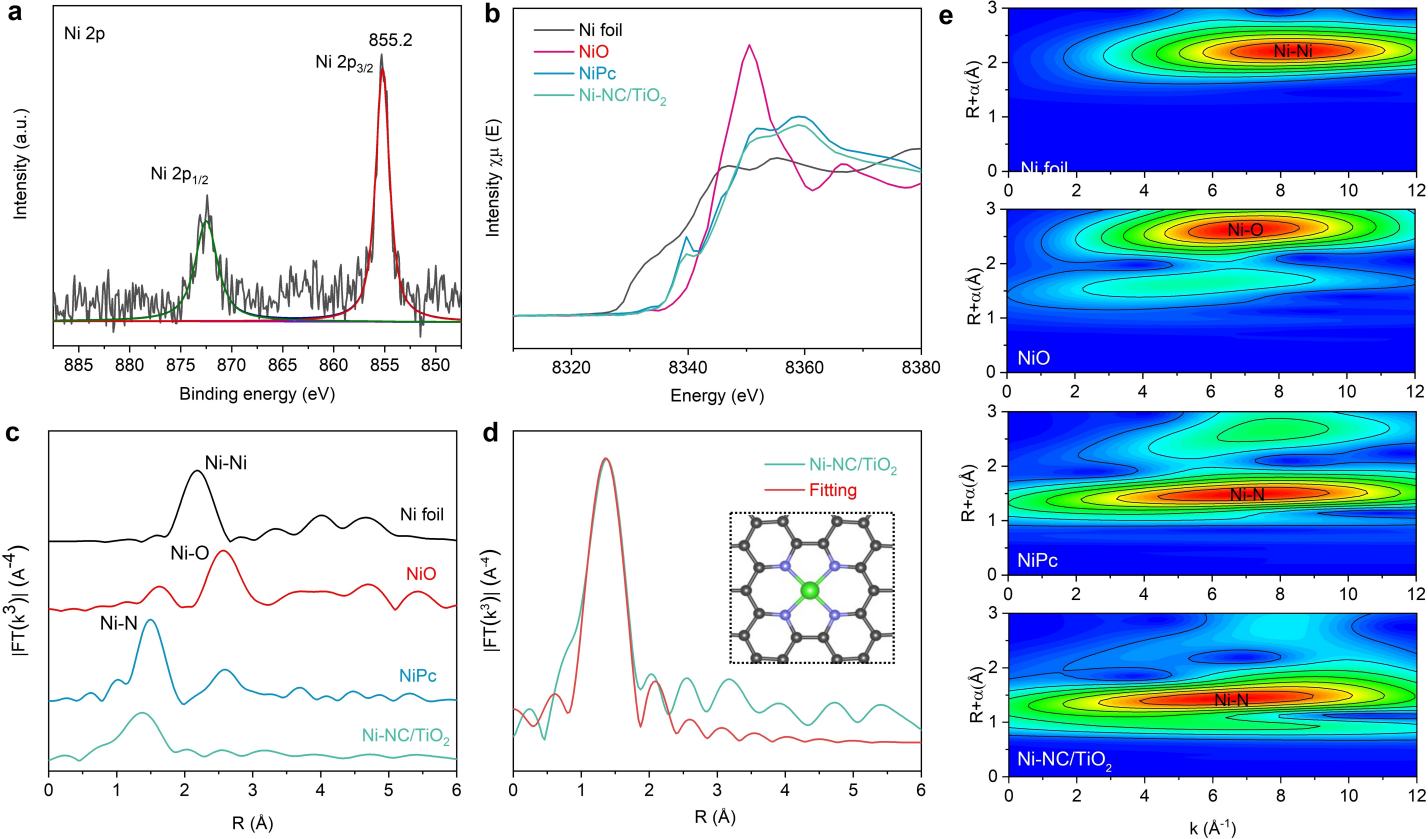
Structural characterizations of Ni−NC/TiO_2_. a) Ni 2p XPS spectrum of Ni−NC/TiO_2_. b) Ni K‐edge XANES spectra. c) FT Ni K‐edge EXAFS spectra of Ni−NC/TiO_2_, Ni foil, NiO and NiPc. The prominent peak of Ni−NC/TiO_2_ is similar to that of NiPc, confirming the presence of Ni−N coordination. d) EXAFS fitting curve of Ni−NC/TiO_2_. The inset is the model Ni−N_4_ structure. Ni, green; N, blue; C, black. The Ni site in Ni−NC/TiO_2_ matches well with a Ni−N_4_ site configuration. e) WT‐EXAFS spectra of Ni foil, NiO, NiPc and Ni−NC/TiO_2_.

The surface compositions and chemical states of Ni−NC/TiO_2_ were investigated by X‐ray photoelectron spectroscopy (XPS). The high‐resolution Ni 2p XPS spectrum of Ni−NC/TiO_2_ (Figure 2a) displays the binding energy of Ni 2p_3/2_ peak at 855.2 eV, which is higher than that of Ni^0^ (853.5 eV) and slightly lower than that of Ni^2+^ (855.8 eV),[^33^, ^34^] suggesting the formation of positively charged Ni species. The high‐resolution N 1s spectrum of Ni−NC/TiO_2_ is deconvoluted into three characteristic peaks at 398.8 eV, 399.7 eV and 400.7 eV (Figure S4), which could be assigned to pyridinic‐N, Ni−N and pyrrolic‐N,^[35]^ respectively. The presence of Ni−N species indicates that Ni atoms are adequately coordinated with N sites.

X‐ray absorption fine structure spectroscopy (XAFS) analysis was further performed to investigate the coordination environment of Ni in Ni−NC/TiO_2_ using Ni foil, NiO and nickel phthalocyanine (NiPc) as references. The Ni K‐edge X‐ray absorption near‐edge structure (XANES) spectra (Figure 2b) show that the absorption edge position of Ni−NC/TiO_2_ is between those of Ni foil and NiO, revealing the cationic Ni sites, in good consistency with the result of XPS analysis. Additionally, Ni−NC/TiO_2_ has a similar pre‐edge profile to NiPc with a peak at 8340 eV, which is attributed to the transition of 1s to 4p and is the feature of planar Ni−N_4_ moiety.^[33]^ Compared with NiPc, the slight decrease in light intensity of Ni−NC/TiO_2_ probably results from the distorted Ni−N_4_ structure. As shown in the Fourier transformed (FT) Ni K‐edge extended XAFS (EXAFS) spectra (Figure 2c), the prominent peak at ca.1.40 Å for Ni−NC/TiO_2_ corresponds to first shell coordination of Ni−N bond,^[36]^ contributing similarly to the NiPc reference, and no obvious Ni−Ni peak at 2.19 Å is detected, revealing the negligible presence of metallic Ni species. These results confirm the presence of atomic dispersion of Ni species in Ni−NC/TiO_2_ in the form of Ni−N coordination, in accordance with the result of dispersed Ni atoms from HAADF‐STEM image. To precisely quantify the coordination microenvironment of Ni site, the curve fitting for EXAFS spectra was performed (Figure 2d, Figure S5, and Table S1). As shown in Figure 2d, the fitting results of the first coordination shell verify that the Ni site in Ni−NC/TiO_2_ is four‐coordinated by N atoms, matching well with a Ni−N_4_ site configuration. In addition, the wavelet transform EXAFS (WT‐EXAFS) spectra (Figure 2e) show that Ni−NC/TiO_2_ and NiPc exhibit similar contour plot with only one intensity maximum at 6.5 Å^−1^ instead of the Ni−Ni interaction (c.a. 8.4 Å^−1^),^[37]^ This further manifests the formation of dispersed Ni atoms with Ni−N coordination. All of above characterizations demonstrate that Ni species are atomically dispersed in Ni−NC/TiO_2_ with Ni−N_4_ moiety.

**Figure 3:**
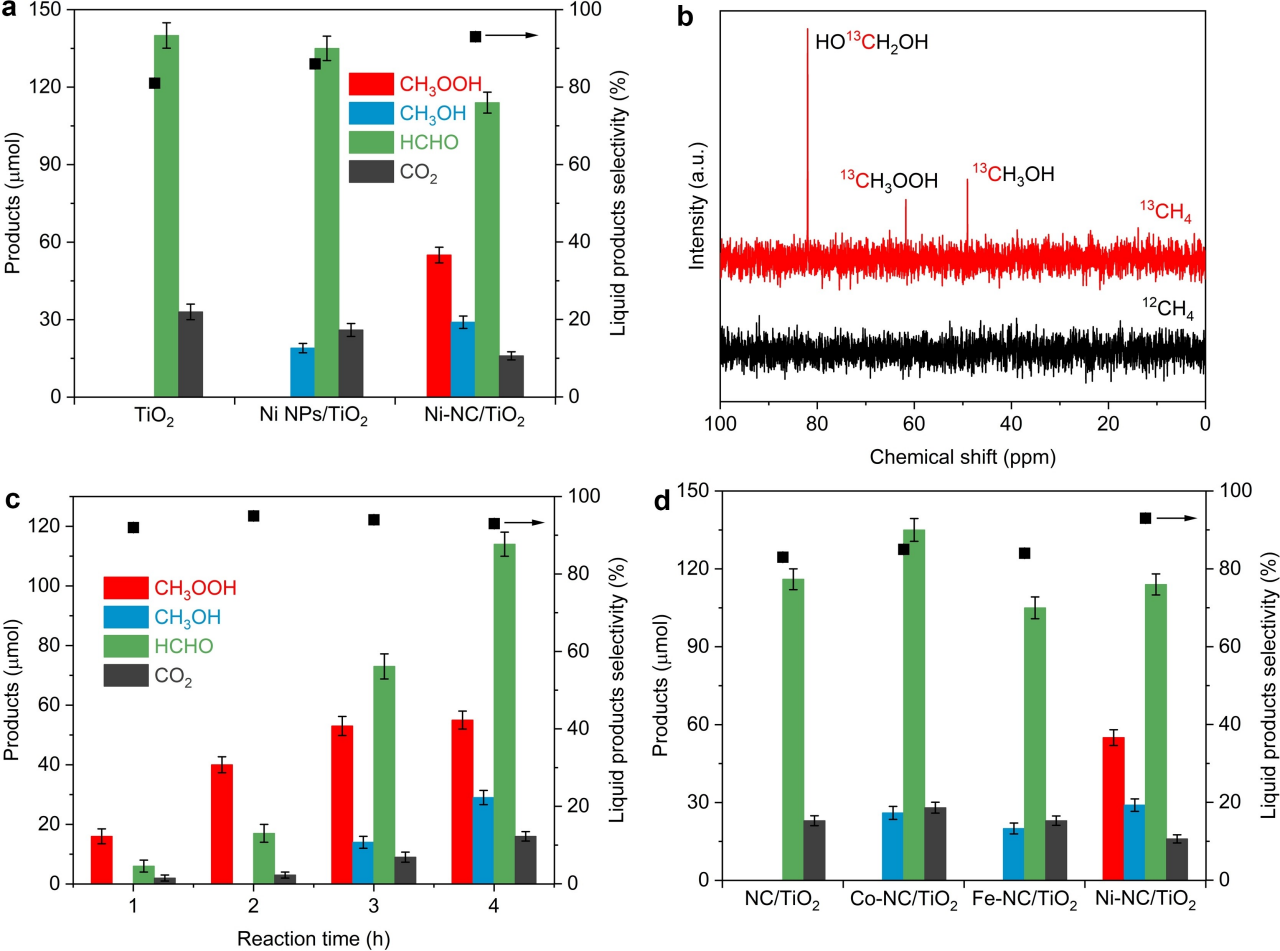
The photocatalytic performance of CH_4_ oxidation with O_2_. a) Yields of oxygenated products and the selectivity of liquid products over TiO_2_, Ni−NC/TiO_2_, and Ni NPs/TiO_2_ after 4 h of light irradiation. b) ^13^C NMR spectrum of liquid products over Ni−NC/TiO_2_ using 0.8 MPa ^13^CH_4_ after 6 h of light irradiation. c) Yields of oxygenated products and the selectivity of liquid products over Ni−NC/TiO_2_ under different irradiation time. d) Yields of oxygenated products and the selectivity of liquid products over different photocatalysts after 4 h of light irradiation. Error bars represent standard deviations of three replicate measurements.

Photocatalytic CH_4_ oxidation performance was evaluated in a batch reactor at room temperature using only O_2_ as the oxidant.[^22^, ^23^] As shown in Figure 3a, only HCHO was detected in the liquid phase over pristine TiO_2_ after 4 h of irradiation with a yield of 140 μmol, accompanied by 33 μmol of CO_2_. For Ni NPs/TiO_2_, the yields of HCHO and CO_2_ slightly decreased to 135 and 26 μmol, respectively, with the production of a small amount of CH_3_OH (19 μmol). The selectivity for C1 oxygenated products increased from ≈81 % over pristine TiO_2_ to ≈86 % over Ni NPs/TiO_2_. Due to CH_3_OH being the precursor of HCHO and CO_2_ in the photocatalytic CH_4_ oxidation,[^22^, ^23^] the trace or small amount of CH_3_OH observed over TiO_2_ and Ni NPs/TiO_2_ suggests the facile overoxidation of CH_4_. By comparison, a much higher yield of primary products CH_3_OOH (55 μmol) and CH_3_OH (29 μmol) together with 114 μmol of HCHO were produced over Ni−NC/TiO_2_, and the amount of CO_2_ decreased to 16 μmol. This leads to a remarkable ≈93 % oxygenates selectivity and the corresponding apparent quantum efficiency (AQE) for oxygenates at 360 nm was determined to be 1.9 %. The yield and selectivity of liquid oxygenates of Ni−NC/TiO_2_ are higher than those of Ni−NPs/TiO_2_ and TiO_2_, demonstrating the superiority of single atom Ni−NC cocatalysts for the photocatalytic CH_4_ oxidation. The excellent photocatalytic performance observed over Ni−NC/TiO_2_ is comparable to or even outperforms most reported photocatalysts decorated with either noble metal or non‐noble metal cocatalysts under similar experiment conditions (Table S2).[^12^, ^13^, ^16^, ^17^, ^19^, ^20^, ^22^, ^23^, ^24^, ^25^, ^38^, ^39^, ^40^]

Reactions without photocatalyst, without light or replacing CH_4_ with Ar did not yield any product. Isotope labelling experiment using ^13^CH_4_ was performed to elucidate the source of carbon atoms of the products. ^13^C NMR spectrum shows three obvious peaks assigned to CH_3_OOH, CH_3_OH and HCHO (Figure 3b), suggesting that the produced oxygenates originated from methane, instead of carbon materials in Ni−NC/TiO_2_. In addition, no liquid products were detected without the introduction of O_2_ (Figure S6), which indicates that O_2_ is the necessary for photocatalytic CH_4_ oxidation. Isotopic experiments with oxygen revealed that O_2_ molecules was the oxygen source of the produced oxygenates (Figure S7). The overall yield of oxygenates was increased with the reaction time, and the formation of CH_3_OH was observed by extending the irradiation time over 3 h (Figure 3c). Increasing the water amount was conducive to promoting the production of oxygenates and suppressing the overoxidation of CH_4_ to CO_2_ (Figure S8). There was marginal loss in the photocatalytic performance and selectivity for oxygenates after consecutive five runs (Figure S9), and the morphology and structure of catalyst remained unchanged (Figures S10 and S11). These results confirm the good stability of Ni−NC/TiO_2_. Increasing the amount of Ni from 0.5 wt % to 1.1 wt % did not noticeably improve the performance of Ni−NC/TiO_2_ (Figure S12), because excessive loading amount of Ni−CN can shield light absorption of TiO_2_ (Figure S13). When Ni was replaced with Co and Fe, the total amounts of oxygenates were reduced, due to no detectable formation of CH_3_OOH (Figure 3d). This demonstrates that the isolated Ni site in Ni−NC with unique properties play an important role for efficient photooxidation of methane to oxygenates.

**Figure 4:**
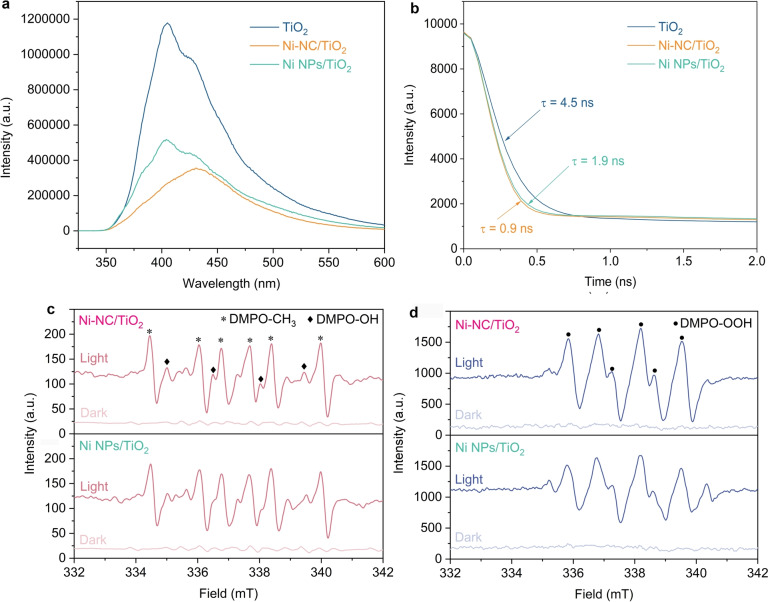
Mechanistic investigation of photocatalytic CH_4_ oxidation. a) PL spectra and b) Time‐resolved PL decay plots of TiO_2_, Ni−NC/TiO_2_, and Ni NPs/TiO_2_. EPR spectra for detecting ⋅CH_3_ radicals (c) and ⋅OOH radicals (d) over Ni−NC/TiO_2_ and Ni NPs/TiO_2_ under dark and light irradiation.

To understand the role of cocatalysts in photocatalytic reaction, the photoluminescence (PL) spectra of the samples were performed to study the photogenerated charge separation efficiency (Figure 4a). Bare TiO_2_ shows an intensive emission peak at 400–440 nm upon excitation at 320 nm. After the introduction of Ni NPs or Ni−NC on TiO_2_, the PL intensity is remarkably decreased, and Ni−NC/TiO_2_ exhibits a lower emission peak than Ni NP/TiO_2_, indicating that Ni−NC favorably prevents the recombination of charge carriers compared to Ni NPs cocatalysts. The time‐resolved PL spectra were carried out to investigate the dynamics of charge carriers (Figure 4b). The average lifetime of Ni−NC/TiO_2_ (0.9 ns) is shorter than those of Ni NP/TiO_2_ (1.9 ns) and TiO_2_ (4.5 ns), in line with the typical cocatalyst/semiconductor systems in which the facile electrons transfer from semiconductors to cocatalysts leads to fast fluorescence decay,[^41^, ^42^] revealing the excellent ability of Ni−NC to accelerate the transfer of photogenerated electrons. These results demonstrate the positive role of Ni−CN in efficiently separating electrons and holes, thereby leading to the enhanced performance of photocatalytic CH_4_ oxidation.

Generally, for photocatalytic CH_4_ aerobic reaction in aqueous solution, the C−H bond of CH_4_ is oxidized by photo‐generated active oxygen species to form ⋅CH_3_ radicals, which would react with oxygen‐derived free radicals to produce oxygenates.^[23]^ To elucidate the reaction mechanism of photocatalytic CH_4_ oxidation, electron paramagnetic resonance (EPR) with 5,5‐dimethyl‐1‐pyrroline‐N‐oxide (DMPO) was conducted. As shown in Figure 4c, ⋅CH_3_ radicals are detected on both Ni−NC/TiO_2_ and Ni NP/TiO_2_, and the intensities of ⋅CH_3_ radicals of Ni−NC/TiO_2_ is slightly higher than that of Ni NP/TiO_2_, revealing that the activation of CH_4_ to ⋅CH_3_ radicals occurs in selective photo‐oxidation of CH_4_ in aqueous solution. Figure S14 shows that strong EPR signals assigned to ⋅OH radicals are observed without the introduction of CH_4_. The decreased intensity of ⋅OH radicals in the presence of CH_4_ may be due to the highly active ⋅OH radicals participating in CH_4_ oxidation, such as deep oxidation of CH_4_ to HCHO and CO_2_.^[22]^ For the intermediates in photocatalytic O_2_ reduction, one set of EPR signals that are assigned to ⋅OOH radicals appear upon illumination (Figure 4d). The observed ⋅OOH radicals are easily produced from O_2_ reduction with protons by photogenerated electrons. Clearly, the signal intensity of ⋅OOH radicals of Ni−NC/TiO_2_ is higher than that of Ni NP/TiO_2_, indicating that Ni−CN cocatalyst can facilitate the formation of ⋅OOH radicals compared with Ni NP cocatalyst. The high amount of ⋅OOH radicals probably lead to the enhanced production of CH_3_OOH and other oxygenates.

Based on the above results, a plausible photocatalytic CH_4_ oxidation mechanism on Ni−NC/TiO_2_ is depicted in Figure S15. Under light irradiadtion, electrons and holes are generated on TiO_2_. The photogeneratred electrons are transferred to single Ni−NC sites to promote the reducion of O_2_ to form ⋅OOH radicals, while the powerful holes are left on the surface of TiO_2_ to initiate the CH_4_ oxidation to produce ⋅CH_3_ radicals. These two radicals can easily combine to form the primary product CH_3_OOH, which can be subsequently transformed into CH_3_OH and HCHO. The single Ni−NC sites guarantee the efficient separation of photogenerated electrons and holes and the favourable formation of ⋅OOH radicals by mild reduction of O_2_, ultimately leading to excellent performance of photocatalytic CH_4_ oxidation with O_2_. To demonstrate this hypothesis, the detailed reaction pathways were calculated by density functional theory (DFT) calculations. The optimized structural models of Ni−NC/TiO_2_ and Ni NPs/TiO_2_ are given in Figure S16.

**Figure 5:**
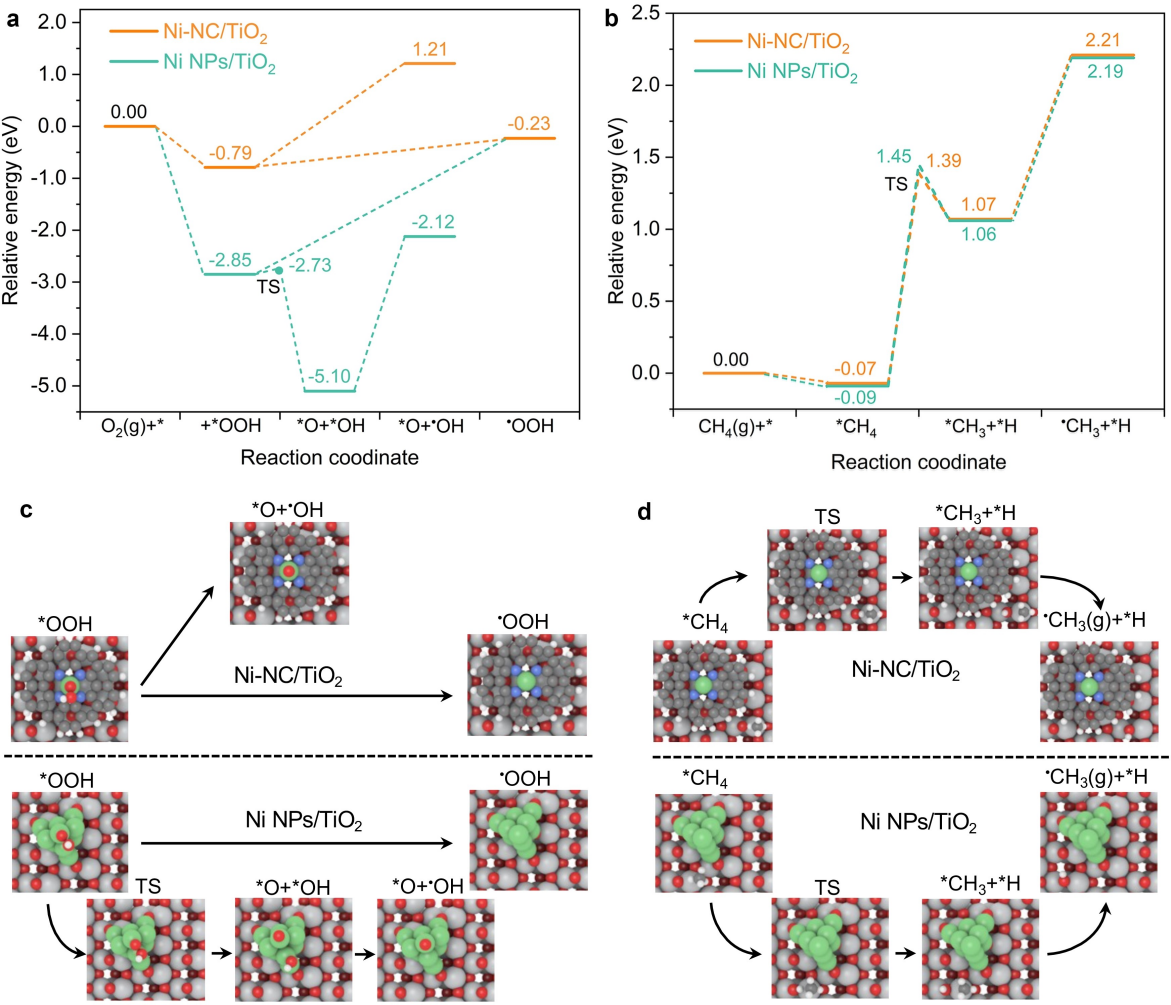
Calculated reaction energy of O_2_ and CH_4_ activation on Ni−NC/TiO_2_ and Ni NPs/TiO_2_. a) Energy profiles of O_2_ reduction to ⋅OOH and ⋅OH radicals. b) Energy profiles of CH_4_ activation to ⋅CH_3_ radicals. c) and d) The structures of reaction intermediates and transition states (TS). Ni, green; N, blue; C, black; O, red; Ti, gray.

The energy profiles of the O_2_ reduction and CH_4_ activation reactions are illustrated in Figure 5a and b, with the corresponding structures of reaction intermediates and transition states shown in Figure 5c and d. The activation of O_2_ to form *OOH species is an exothermic reaction on Ni−NC/TiO_2_ and Ni NP/TiO_2_, with a reaction energy of −0.79 and −2.85 eV, respectively. The comparatively unfavourable formation of *OOH species indicates the weak surface adsorption of Ni−NC because of its unique electronic structure. This results in the preferential desorption of *OOH species to generate ⋅OOH radicals that can participate in the production of CH_3_OOH, instead of the subsequent dissociation of *OOH to form ⋅OH radicals due to the large reaction energy (2.00 eV). By contrast, the desorption energy of *OOH species on Ni NPs is as high as 2.62 eV. Compared with *OOH desorption, the dissociation of *OOH to *O+*OH is more preferred on Ni NPs, with a reaction energy of −2.25 eV and an energy barrier of 0.12 eV, and the produced *OH species on Ni NPs could desorb to form ⋅OH radicals for oxidizing oxygenates to CO_2_. These results indicate that Ni−NC cocatalyst is beneficial for the production of ⋅OOH radicals in O_2_ reduction, consistent with the EPR results, which contributes to the production of oxygenates. The different behaviors of Ni NPs and Ni−NC on O_2_ activation probably because *OOH is very unstable on metallic Ni NPs and is easily dissociated to form strong Ni−O bonds, as indicated by the large reaction energy (−2.25 eV), while Ni−NC is stabilized by N coordination, thus unfavorable for further dissociation of *OOH.

For CH_4_ activation, the reaction energy for the cleavage of the first C−H bond of 1.14 eV on Ni−NC/TiO_2_ is quite similar to that on Ni NPs/TiO_2_ (1.15 eV), with a relatively lower energy barrier (1.46 eV vs. 1.54 eV). Likewise, the reaction energies for the subsequent *CH_3_ desorption to ⋅CH_3_ radicals on Ni−NC/TiO_2_ and Ni NPs/TiO_2_ are similar (1.14 eV vs. 1.13 eV). It clearly shows that there is no significant difference in the activation of methane over Ni−NC and Ni NPs cocatalysts. As a result, the different pathways of O_2_ reduction over Ni−NC/TiO_2_ and Ni NP/TiO_2_ primarily contribute to the differences of activity and selectivity in photocatalytic CH_4_ oxidation.

## Conclusion

In summary, atomically dispersed Ni−NC/TiO_2_ has been developed by a facile one‐pot solvothermal method for room‐temperature photocatalytic CH_4_ oxidation with O_2_ to C1 oxygenates with 93 % selectivity. The single‐atom Ni−NC sites function as electron capture centers to achieve efficient separation of charge carriers in Ni−NC/TiO_2_. Moreover, the isolated Ni atoms are active for the favorable formation and desorption of ⋅OOH radicals in O_2_ reduction, rather than being active for the production of ⋅OH radicals that are more likely to facilitate the overoxidation of oxygenates to CO_2_. Such unique properties of Ni−NC results in a prominent C1 oxygenates productive rate and high selectivity. This work is the first case of single metal atoms anchored N‐doped carbon material as a cocatalyst to promote the performance of photocatalytic aerobic oxidation of CH_4_, which may drive the discovery of more earth‐abundant and low‐cost photocatalysts for efficiently and selectively oxidizing CH_4_ to solar fuels and chemicals.

## Conflict of interest

The authors declare no conflict of interest.

1

## Supporting information

As a service to our authors and readers, this journal provides supporting information supplied by the authors. Such materials are peer reviewed and may be re‐organized for online delivery, but are not copy‐edited or typeset. Technical support issues arising from supporting information (other than missing files) should be addressed to the authors.

Supporting Information

## Data Availability

The data that support the findings of this study are available from the corresponding author upon reasonable request.
