# Supplementary material for: Atomically Dispersed Nickel Anchored on a Nitrogen‐Doped Carbon/TiO2 Composite for Efficient and Selective Photocatalytic CH4 Oxidation to Oxygenates
Source: Angew Chem Int Ed Engl. 2022 Dec 16;62(4):e202215057. doi: 10.1002/anie.202215057 (PMC10107830; doi:10.1002/anie.202215057)
Supplement: Supplementary file 1 — Supporting Information [file ANIE-62-0-s001.pdf]

Angewandte Chemie
Eine Zeitschrift der Gesellschaft Deutscher Chemiker

Supporting Information

Atomically Dispersed Nickel Anchored on a Nitrogen-Doped Carbon/TiO_2 Composite for Efficient and Selective Photocatalytic CH_4 Oxidation to Oxygenates

H. Song, H. Huang, X. Meng, Q. Wang, H. Hu, S. Wang, H. Zhang, W. Jewasuwan, N. Fukata, N. Feng*, J. Ye**

Experimental methods

Chemicals and materials. Commercial TiO_2 (AEROXIDE TiO_2 P25, Lot No. 614041498) was supplied from Evonik-Degussa. $NiCl_2\cdot 6H_2O$, $CoCl_2\cdot 6H_2O$, $FeCl_3\cdot 9H_2O$ and formamide TiO_2 were purchased from Wako. All materials were used as received without further purification.

Synthesis of catalyst. Ni-NC/TiO_2 was synthesized using a previously reported method with a slight modification.[1] In brief, 500 mg of TiO_2 (P25) and 50 mg of $NiCl_2\cdot 6H_2O$ was added into 15 mL of formamide. After stirring for 30 min, the mixture was added into a 50 mL Teflon-line stainless autoclave and kept at 180 °C for 12 h. Then, the precipitate was collected by centrifugation, washed with diluted HNO_3 solution and water for several times. Finally, the sample was dried at 80 °C under vacuum for 12 h, and further calcined in Ar atmosphere at 200 °C for 2 h to obtain Ni-NC/TiO_2. Fe-NC/TiO_2 and Co-NC/TiO_2 were synthesized using the similar method, except that $NiCl_2\cdot 6H_2O$ was replaced with $FeCl_3\cdot 6H_2O$ or $CoCl_2\cdot 6H_2O$. For comparison, TiO_2 loaded with Ni nanoparticles was prepared using the incipient wetness impregnation method. 500 mg of TiO_2 and 11 mg of $NiCl_2\cdot 6H_2O$ were dispersed into 20 mL of water and dried at 60 °C for 12 h. Then the sample was calcined in air at 400 °C for 4 h and further reduced by H_2 at 400 °C for 1 h. The obtained catalyst was denoted as Ni NPs/TiO_2.

Photocatalytic activity measurements

Photocatalytic CH_4 oxidation performance was evaluated with a method similar to our previously reported work. Briefly, 180 mL water containing 10 mg photocatalyst was added into a batch reactor equipped with a quartz window to allow light irradiation. Then, 0.1 MPa O_2 and 2 MPa CH_4 (or 0.8 MPa $^{13}CH_4$) were added successively to the batch reactor. The light source was provided by a 300 W Xe lamp (300 nm < λ < 500 nm). A water bath was used to control the reaction temperature at 25 °C. After the reaction, the reactor was placed in the refrigerator to a temperature below 10 °C. After that, the gas product was collected and the amounts of CO_2 and CH_4 in the gas product were analyzed by a gas chromatograph equipped with methanizer and flame ionization detector (FID). CH_4 and CO_2 can be separated and their peaks appeared at different retention time. The amounts of CO_2 and CH_4 were quantified by comparing the peak area of CO_2 and CH_4 against calibration curves. The liquid solution was obtained by centrifugation. The amount of CH_3OOH and CH_3OH in the liquid product were analyzed by nuclear magnetic

resonance spectroscopy and the amount of HCHO was analyzed by colorimetric method. The amounts of CH_3OOH and CH_3OH in the liquid were quantified by ^1H NMR (JEOL ECS 400 MHz). Typically, 0.5 mL of liquid product was mixed with 0.1 mL of dimethyl sulfoxide (DMSO)/D_2O solution (the volume ratio of DMSO to D_2O is 1/2000, and DMSO was used as an internal standard). The concentrations of standard product solutions were plotted versus the area ratio of product to DMSO for obtaining calibration curves. The products were quantified by comparing the peak area of product and internal standard in ^1H NMR against calibration curves. The amount of HCHO was quantified using a colorimetric method[2]. Firstly, 100 mL of reagent aqueous solution was prepared by dissolving 15 g of ammonium acetate, 0.3 mL of acetic acid and 0.2 mL of pentane-2,4-dione in water. Then, 0.5 mL of liquid product was mixed with 2.0 mL of water and 0.5 mL of reagent solution. The mixed solution was heated at 35 °C in a water bath for around 30 min. Then the absorption intensity of the obtained solution at 412 nm was measured by a UV-Vis absorption spectroscopy. The amount of HCHO in the liquid product was determined by the standard curve.

Characterization

An X-ray diffractometer with Cu Kα radiation (PANalytical B.V., Netherlands) was used to acquire the XRD spectra of samples. The loading amounts of Ni were characterized with a inductively coupled plasma optical emission spectrometry (ICP-OES, HORIBA). Transmission electron microscope (JEM-2100F, JEOL, Japan) was employed to obtain the TEM and HRTEM images. STEM and the corresponding EDX elemental mapping images were characterized using a JEM-ARM200F field-emission transmission electron microscope. X-ray photoelectron spectra (XPS) were collected by an electron spectrometer (Escalab 250 Thermo scientific, America, Al Kα, 1486.6 eV), and the data were calibrated with the C 1s at 284.8 eV of surface carbon adventitious. X-ray absorption fine structure (XAFS) spectra of Ni K- edge were carried out on the BL14W1 beamline of Shanghai Synchrotron Radiation Facility, China. XAFS spectra of samples were obtained under the fluorescence mode with a 32-element Ge solid state detector. Ni foil, NiO and nickel phthalocyanine (NiPc) were used as reference samples. The Athena and Artemis software was used to analyzed the data. Electron paramagnetic resonance (EPR) spectroscopy measurement was conducted on Magnettech MS-5000X at room temperature using 5, 5-Dimethyl-1-pyrroline-*N*-oxide (DMPO) as the radical trap. The photoluminescence (PL) spectra and time-resolved PL spectra were obtained under the excitation of 320 nm with a

spectrofluorometer (Fluorolog-3, Horiba Jobin Yvon, USA). Reflectance spectra of catalysts were measured using an ultraviolet-visible spectrophotometer (UV-2600, Shimadzu, Japan) with 100 mg of samples, and then the resulting reflectance spectra were converted into ultraviolet-visible absorption spectra using the Kubelka-Munk function.

Computation details

Computational models

The anatase $TiO_2(101)$ surface was represented by a (2 × 5) slab with two O–Ti–O layers, in which the bottom layer is fixed. This surface model contains 80 Ti and 160 O atoms with a 20 Å vacuum layer and the lattice parameters were a = 20.3999, b = 18.9254, c = 25.8664 Å, α = β = γ = 90°. Such a large model is enough to accommodate a single-atom Ni cocatalyst ($H_{16}C_{36}N_4Ni$) coordinated by four N atoms supported by a H-capped graphene-like fragment and reduce the interactions between neighbouring adsorbed clusters. Another tetrahedron Ni_{10} cluster cocatalyst cut from the face-centered cubic lattice of bulk metallic nickel was obtained as a comparison. The catalytic effects of a photogenerated electron and hole are modeled by introducing an excess H and OH into the slabs, respectively.[3,4] The four optimized computational models are provided in Figure S16.

Density functional theory parameters

All spin-polarized theoretical simulations were performed using the Kohn–Sham formalism with the generalized gradient approximation (GGA) and Perdew-Burke-Ernzerhof (PBE) exchange-correlation functional, as implemented in the VASP code.[5,6] The plane-wave basis sets of 400 eV cutoff kinetic energy to approximate the valence electron densities and the projector augmented wave (PAW) method to account for the core−valence interaction.[7] The Γ-point approximation is employed for the Brillouin zone integration because of the significant size of the supercell. All the structures were relaxed until the forces on each ion were less than 0.05 eV/ Å, and the convergence criterion for energy was set as 10^{-5} eV. Gaussian smearing was used for the electronic states of our models and the width was set to 0.05 eV. The DFT-D3 method of Grimme was used to describe van der Waals interactions,[8] to obtain a better description of the interactions between molecules. The transition states during the reaction pathway were evaluated by the climbing-image nudged elastic band (CI-NEB) combined with minimum-mode following dimer method.[9-11] All the

transition state structures were identified by vibrational frequency analysis. To consider the solvent effect of water, the solvent model (VASPsol) was adopted.[12]

The adsorption energy obtained directly from DFT calculations for adsorbate on the surface models was calculated as:

$$\Delta E_{ads} = E(\text{Adsorbate/Surface}) - E(\text{Adsorbate}) - E(\text{Surface})$$

where E is the total energy calculated using DFT. The zero-point energy (ZPE) correction (E_{ZPE}) was included as:

$$E_{ZPE} = \sum_{i=1}^{number\ of\ models} \frac{1}{2} h\nu_i$$

where vi is DFT obtained vibrational frequency for each intermediate.

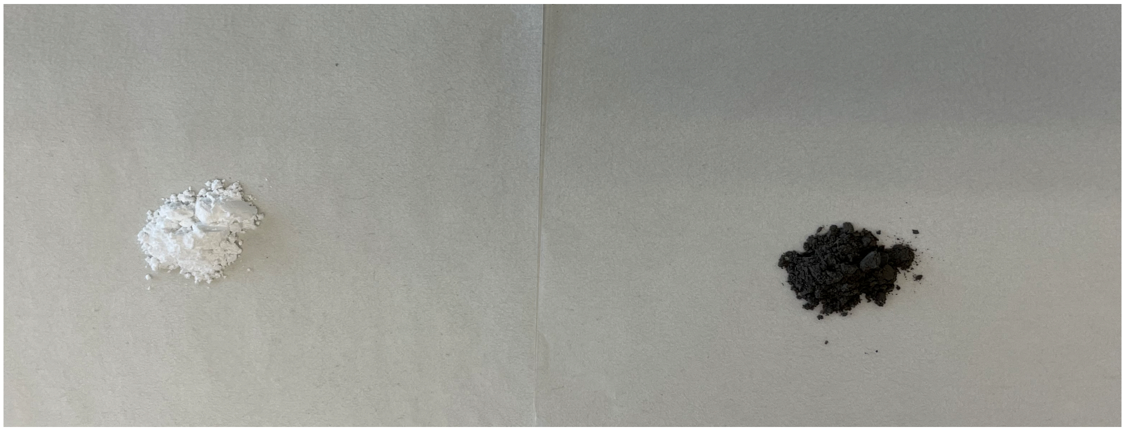

Figure S1. Images of TiO_2 (left) and Ni-NC/TiO_2 (right).

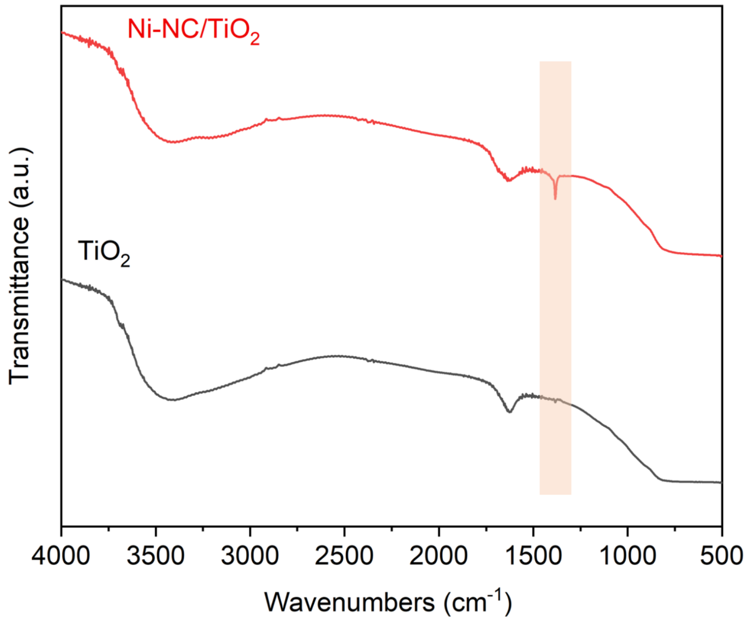

Figure S2. FT-IR spectra of TiO_2 and Ni-NC/TiO_2.

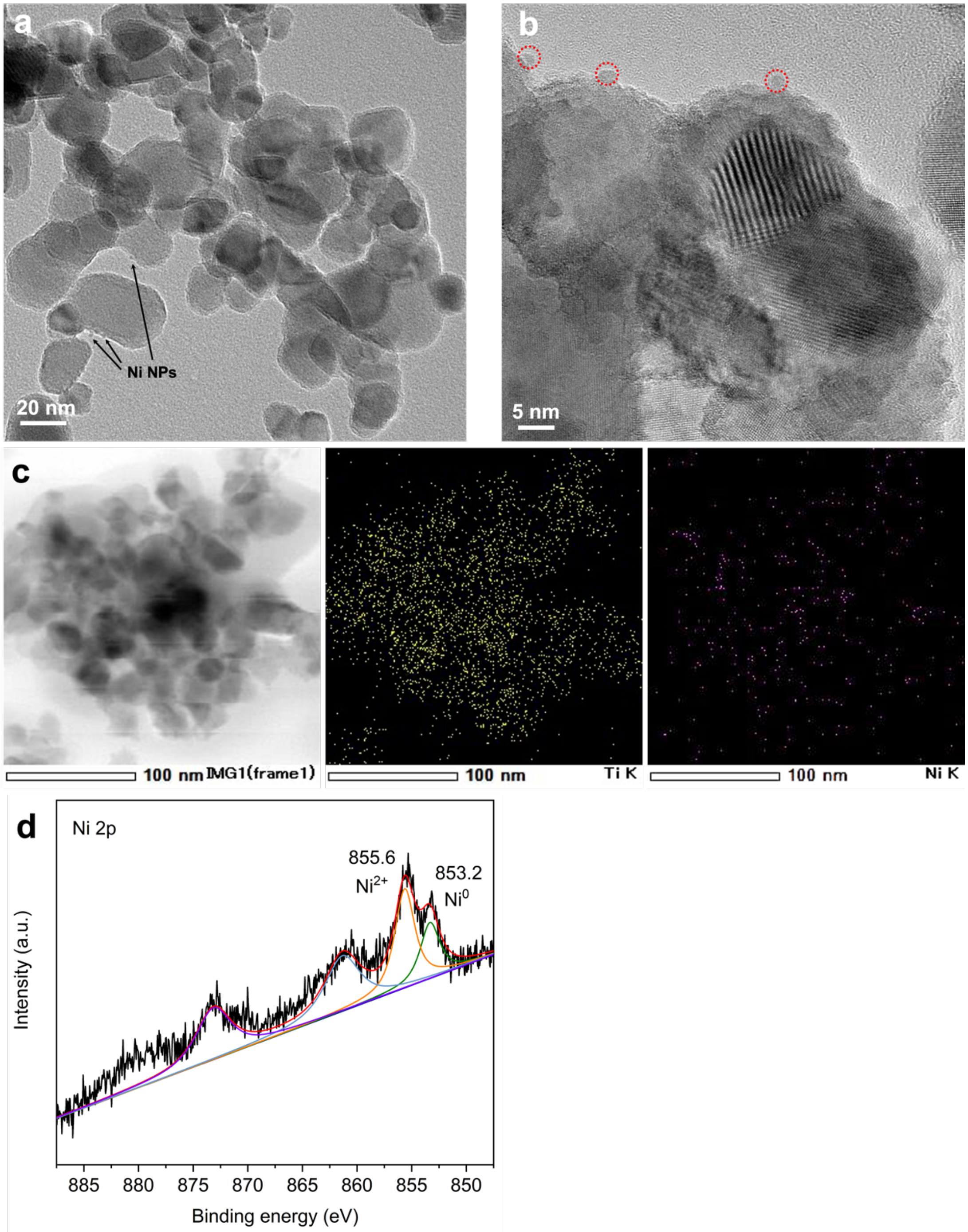

Figure S3. a) TEM image, b) HRTEM image, c) EDX elemental mapping images, and d) Ni 2p XPS spectrum of Ni NPs/TiO_2. Figure S3a and S3b show that small Ni NPs with size of 2~3 nm were formed on TiO_2 surface in Ni NPs/TiO_2. The Ni NPs are marked by red circles in Figure S3b. Figure S3c shows that that Ni element is dispersed on TiO_2. Figure S3d shows that both Ni^0 and Ni^{2+} are present. The existence of Ni^{2+} could be ascribed to the oxidization of catalysts exposed in the air.

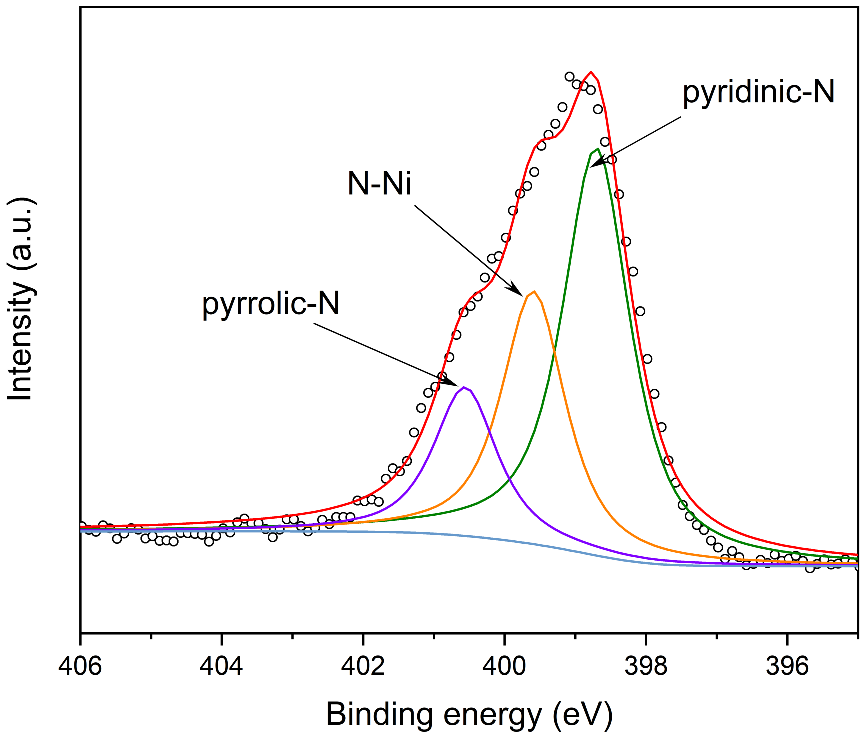

Figure S4. N 1s XPS spectrum of Ni-NC/TiO_2.

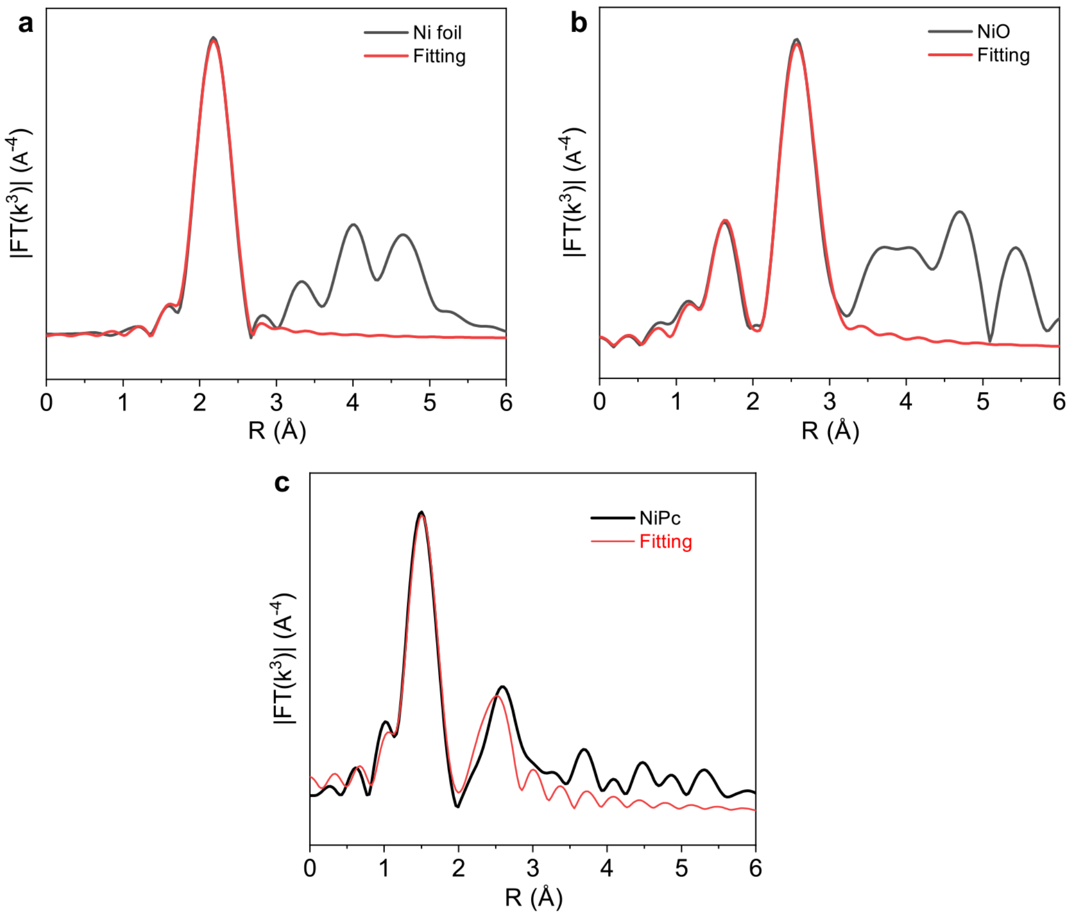

Figure S5. EXAFS fitting curve of a) Ni foil, b) NiO, and c) NiPc.

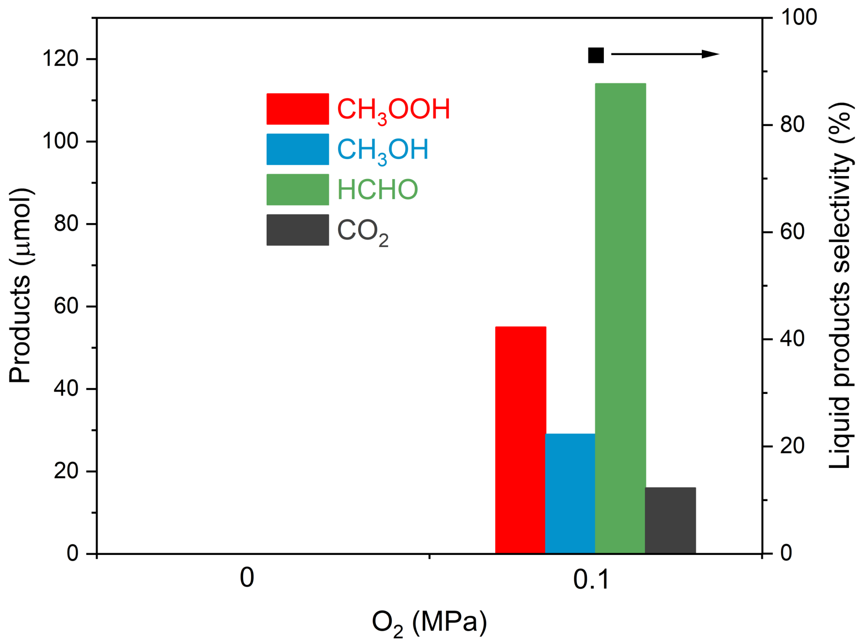

Figure S6. Photocatalytic CH_4 oxidation performance of Ni-NC/TiO_2 for 4 h without and with O_2 (0.1 MPa).

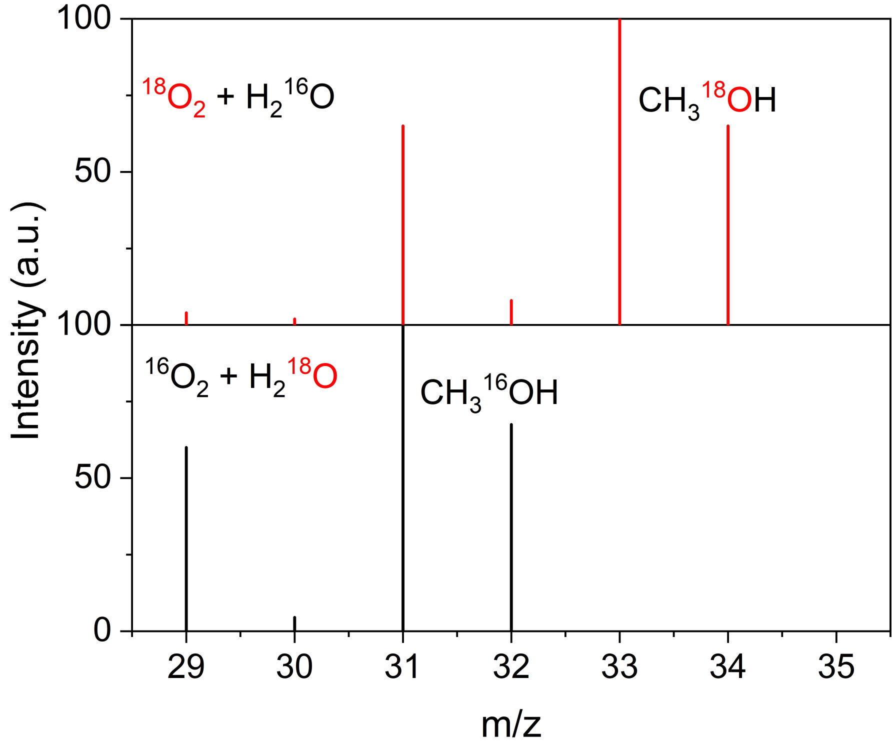

Figure S7. GC-MS spectra of CH_3OH generated over Ni-NC/TiO_2 with $^{18}O_2$ + $H_2{}^{16}O$ or $^{16}O_2$ + $H_2{}^{18}O$ in photocatalytic CH_4 oxidation. Before the measurement, the produced CH_3OOH in the liquid product was first reduced to CH_3OH with $NaBH_4$. As shown in Figure S7, when $^{18}O_2$ and $H_2{}^{16}O$ was used, $CH_3{}^{18}OH$ were detected as the major product. When the reactants were replaced with $^{16}O_2$ and $H_2{}^{18}O$, only the MS signals of $CH_3{}^{16}OH$ was observed. These results indicates that O_2 molecules is the oxygen source of the produced CH_3OOH and CH_3OH. Due to the relatively low sensitivity of GC-MS toward HCHO, we cannot analyze the oxygen source of HCHO. However, it is widely studied that HCHO is a main product in photocatalytic CH_3OH dehydrogenation without the removal of O atoms[13]. Therefore, it can be concluded that the oxygen atoms of produced oxygenates come from O_2.

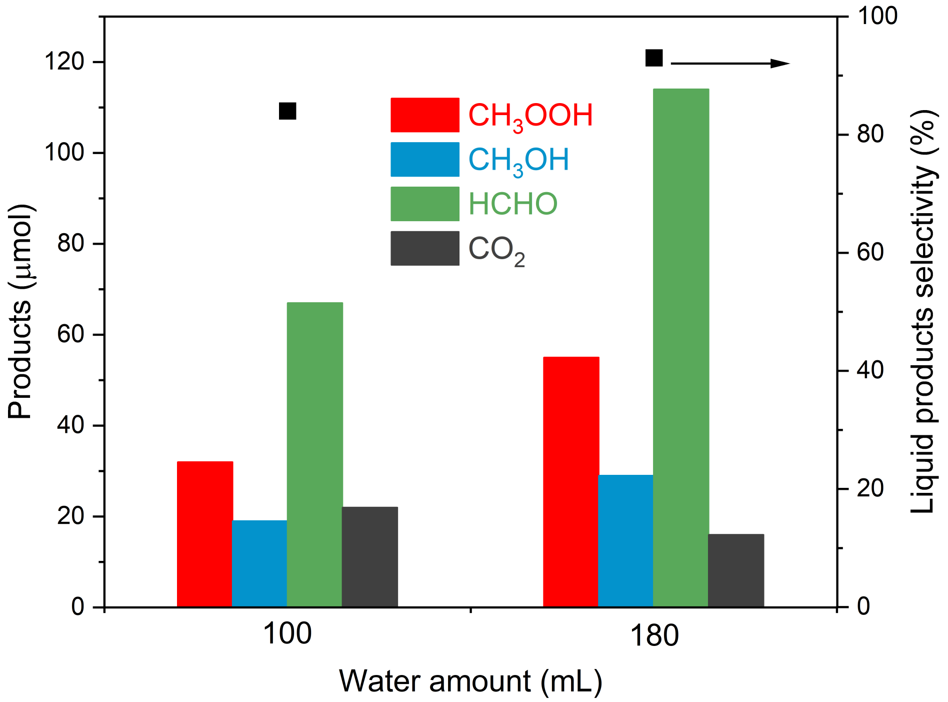

Figure S8. Photocatalytic CH_4 oxidation performance of Ni-NC/TiO_2 for 4 h with different amounts of water.

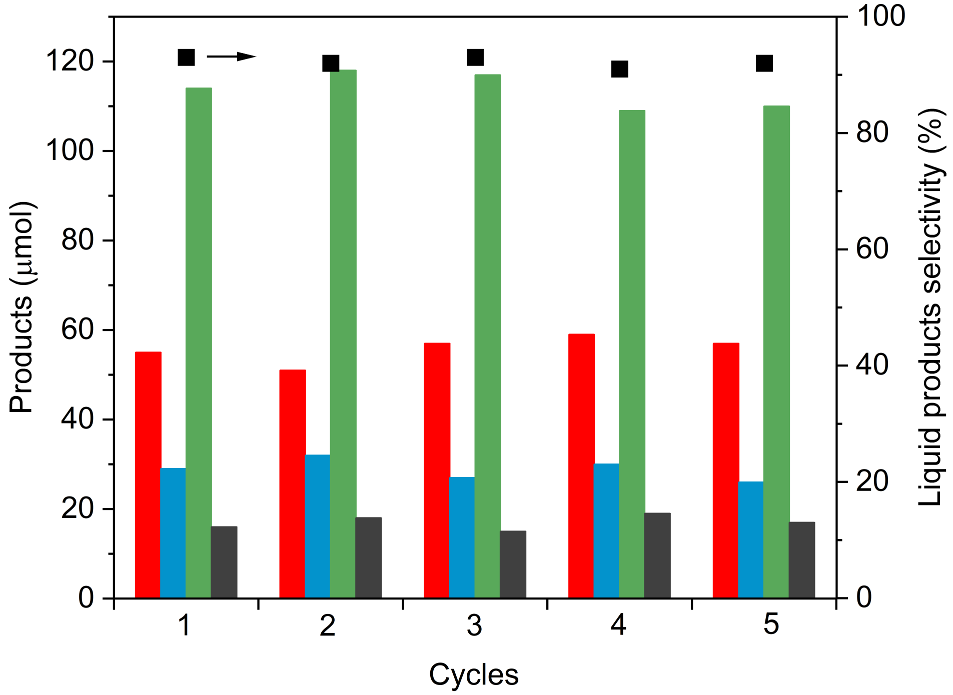

Figure S9. Stability test of Ni-NC/TiO_2 during five photocatalytic CH_4 oxidation cycles. Each cycle was conducted for 4 h.

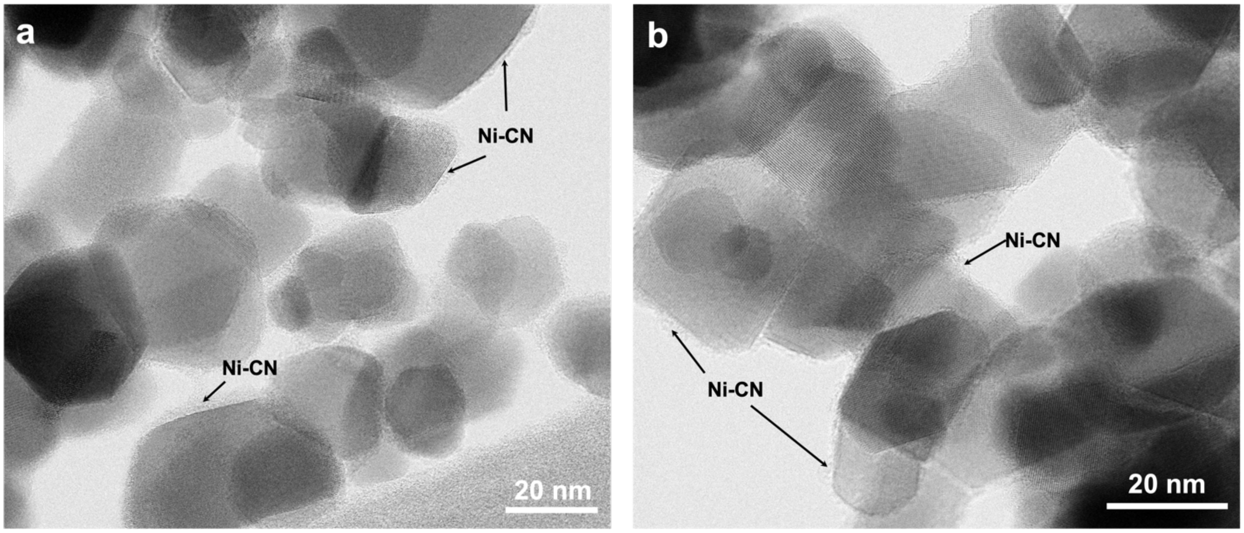

Figure S10. TEM images of Ni-NC/TiO_2 (a) before and (b) after reaction.

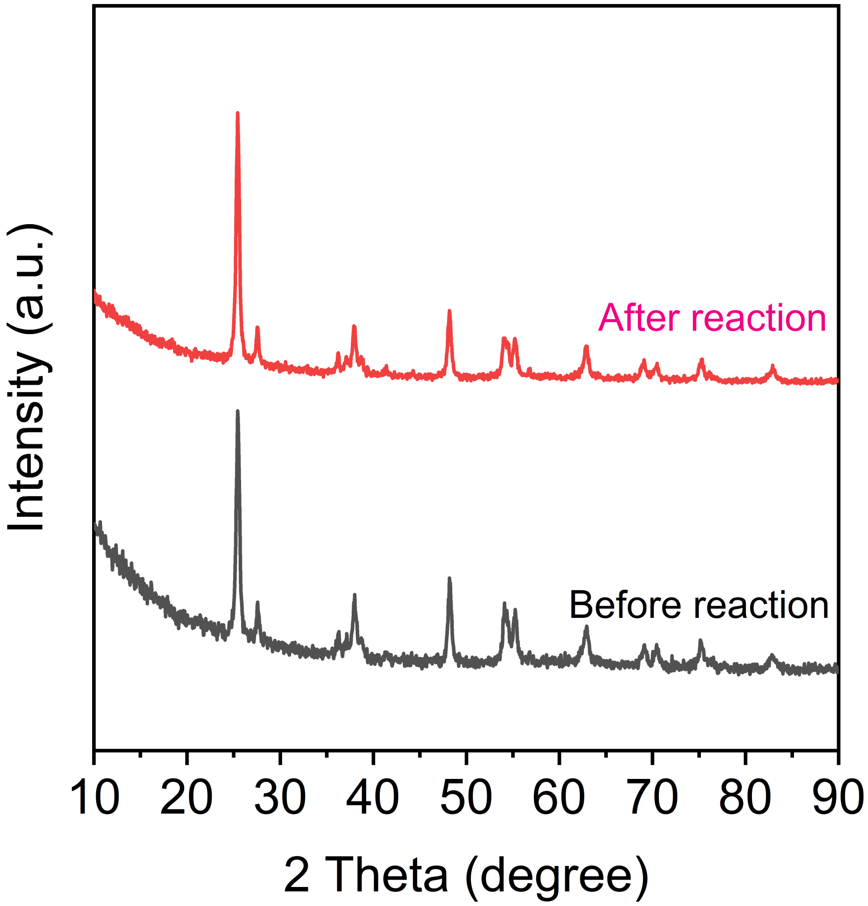

Figure S11. XRD patterns of Ni-NC/TiO_2 before and after reaction.

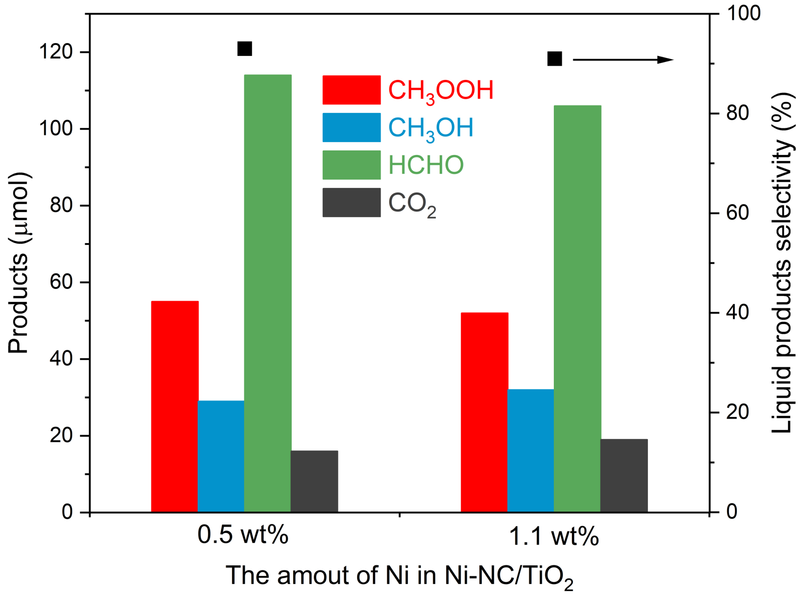

Figure S12. Photocatalytic CH_4 oxidation performance of Ni-NC/TiO_2 with different amounts of Ni.

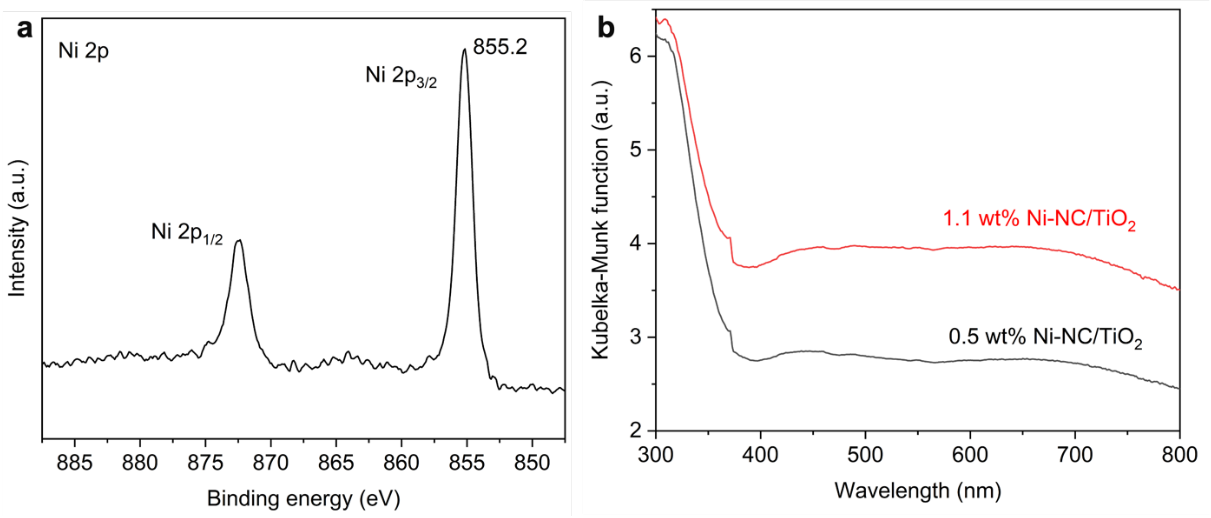

Figure S13. a) Ni 2p XPS spectrum of 1.1 wt% Ni-NC/TiO_2. b) Ultraviolet-visible absorption spectra of 0.5 wt% Ni-NC/TiO_2 and 1.1 wt% Ni-NC/TiO_2. Figure S13a shows that the chemical state of the Ni sites of 1.1 wt% Ni-NC/TiO_2 is same to that of 0.5 wt% Ni-NC/TiO_2. As shown in Figure S13b, 1.1 wt% Ni-NC/TiO_2 showed stronger light absorption than 0.5 wt% Ni-NC/TiO_2, suggesting that 1.1 wt% Ni-NC/TiO_2 contained more CN covered on TiO_2, which can prevent the light absorption of TiO_2 and reduce the generation of photogenerated electrons and holes. Therefore, CH_4 conversion was not significantly increased, although the amount of Ni was increased from 0.5 wt% to 1.1 wt% with no change in chemical state.

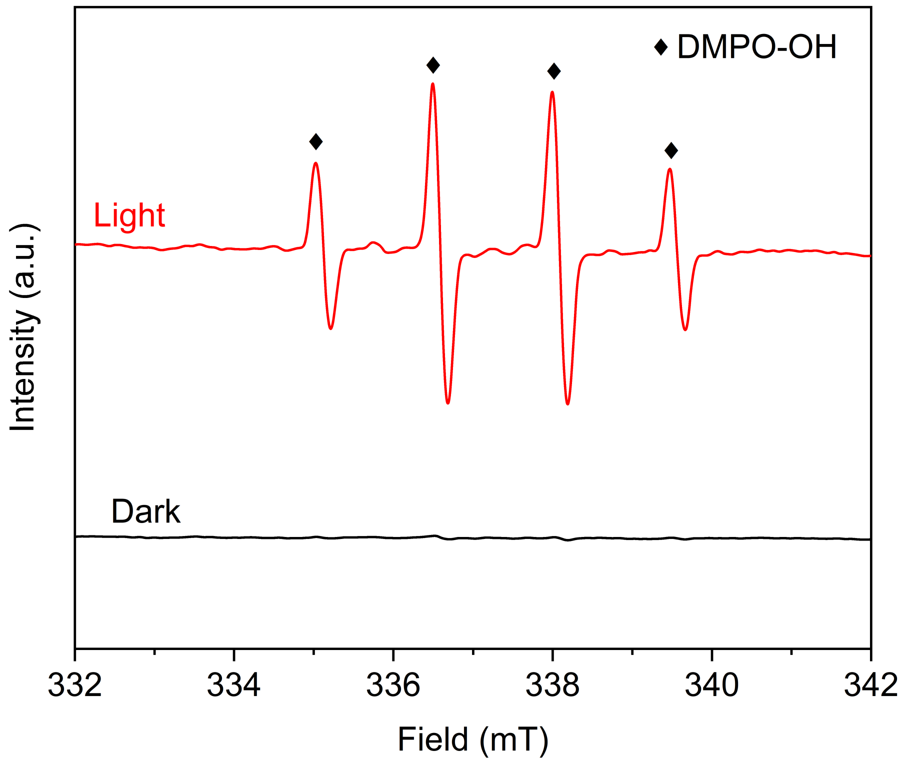

Figure S14. EPR spectra for detecting $^{\bullet}OH$ radicals over Ni-NC/TiO_2 without the introduction of CH_4.

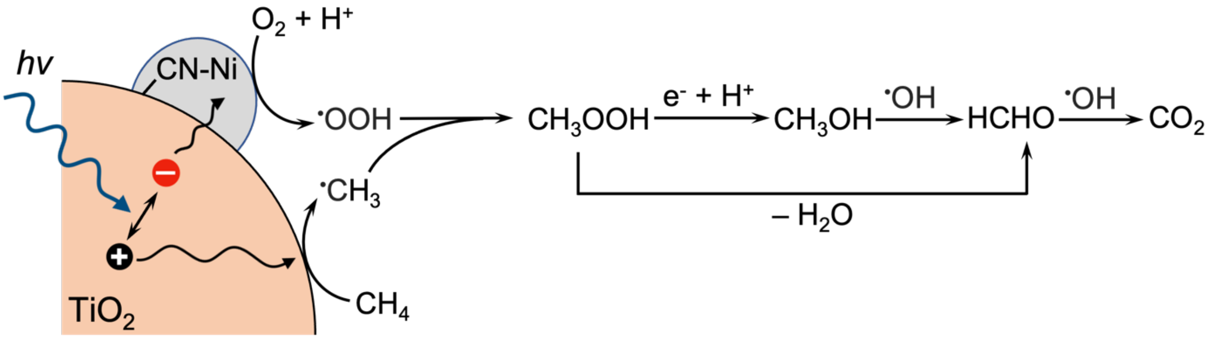

Figure S15. Schematic illustration of the mechanism of photocatalytic CH_4 oxidation with O_2 in water over Ni-NC/TiO_2. The absence of CH_3OOH over TiO_2, NC/TiO_2, Co-NC/TiO_2, Fe-NC/TiO_2, and Ni NPs/TiO_2 is likely due to the fact that they show a higher ability to activate O_2 to form *O, *OH or •OH, thus resulting in CH_3OH to HCHO as the final products. By contrast, Ni-NC/TiO_2 shows a mild reductive ability and *OOH could be preserved (as demonstrated in Figure 5), thus leading to the production of CH_3OOH.

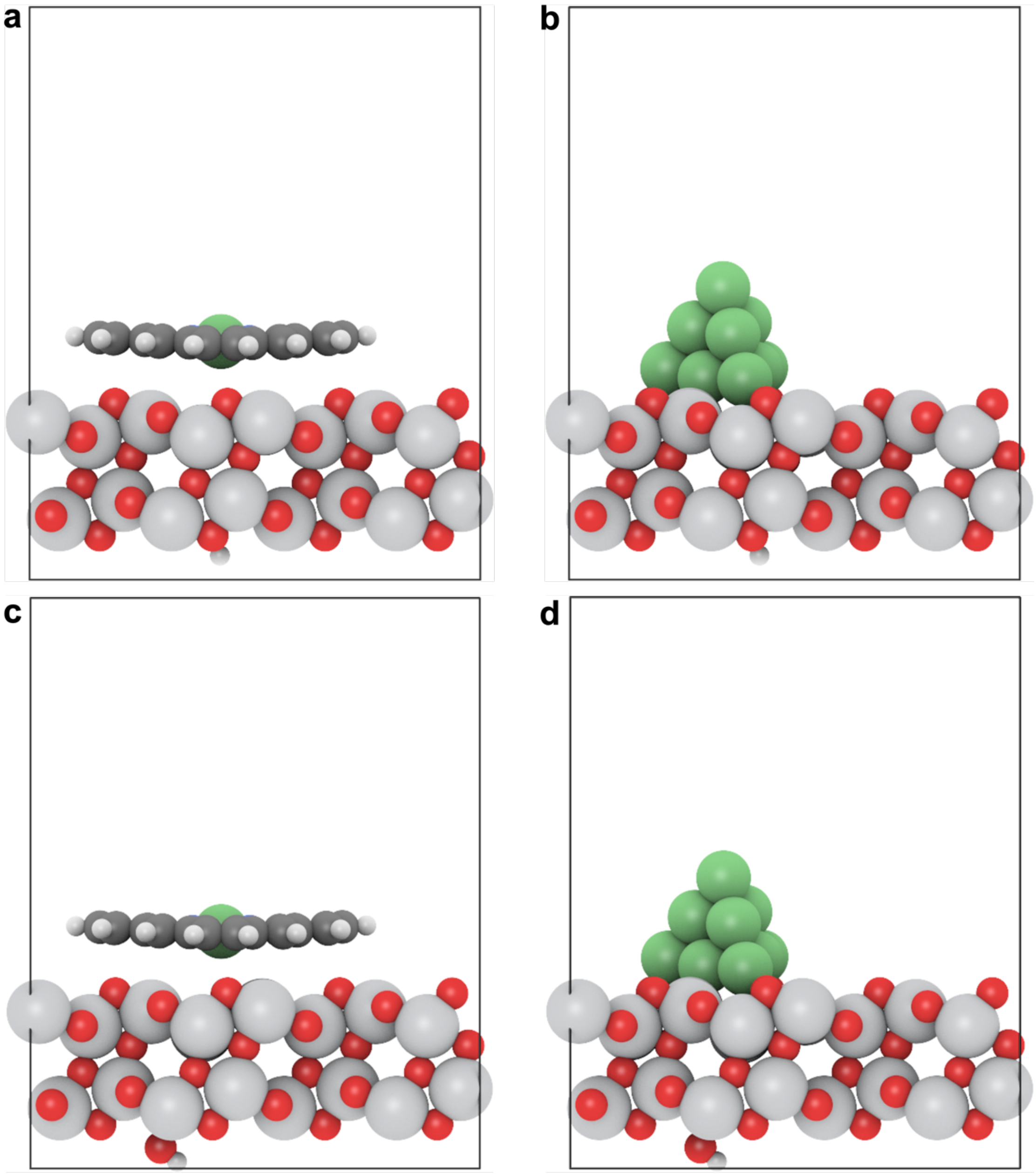

Figure S16. The optimized structural models of a) Ni-NC/TiO_2 with an excess H, b) Ni NPs/TiO_2 with an excess H, c) Ni-NC/TiO_2 with an excess OH and d) Ni NPs/TiO_2 with an excess OH. An excess H or OH is introduced into the slab to model a photogenerated electron or hole, respectively. The models a) and b) were used for the calculation of O_2 activation. The models c) and d) were used for the calculation of CH_4 activation.

Table S1. The fitting EXAFS data for different Ni-based catalysts.

Sample	Shell	CN^a	$R(Å)^b$	$\Delta\sigma^2*10^3$ $(Å^2)^c$	R-factor $(\%)^d$
Ni foil	Ni-Ni	12.0	2.49	7.3	0.008
NiO	Ni–O	6.0	2.09	5.8	0.009
	Ni-Ni	12.0	2.95	6.4	
Ni-NC/TiO_2	Ni-N	3.8	1.85	5.3	0.06
NiPc	Ni-N	4.0	1.91	3.0	0.03
	Ni-C	8.0	2.92	4.7	

[a]CN: coordination number, the Ni-Ni coordination numbers in Ni foil was fixed as 12;
[b]R: distance between absorber and backscatter atoms;
[c]$\Delta\sigma^2$: Debye–Waller factor to account for both thermal and structural disorders;
[d]R factor: goodness of the fit.

Table S2. Comparison of performance in photocatalytic methane oxidation to oxygenates.

Entry	Catalyst	Reaction condition	Liquid oxygenates		Ref.
			Amount (μmol)	Selectivity (%)	
1	Ni-NC/TiO_2	300 W Xe lamp (300-500 nm, 450 mW cm^{-2}), 10 mg catalyst, 2.0 MPa CH_4, 0.1 MPa O_2, 25 ºC, 4 h	198	93	This work
2	Au-CoO_x/TiO_2	300 W Xe lamp (300-500 nm, 450 mW cm^{-2}), 10 mg catalyst, 2.0 MPa CH_4, 0.1 MPa O_2, 25 ºC, 4 h	73	91	*ACS Catal.* 2020, 10, 14318-14326
3	Au/ZnO	300 W Xe lamp (300-500 nm, 450 mW cm^{-2}), 10 mg catalyst, 2.0 MPa CH_4, 0.1 MPa O_2, 25 ºC, 4 h	333.6	92	*J. Am. Chem. Soc.* 2019, 141, 20507-20515
4	3.2%Ag/TiO_2 (001)	300 W Xe lamp (300-500 nm, 450 mW cm^{-2}), 10 mg catalyst, 2.0 MPa CH_4, 0.1 MPa O_2, 25 ºC, 4 h	198	90	*Nat. Commun.* 2022, 12, 4652
5	HSiMo/TiO_2	300 W Xe lamp with a standard AM 1.5G filter, 20 mg catalyst, 3.0 MPa CH_4, 2.0 MPa O_2, 150 ºC, 2 h	~60	82.4	*J Mater Chem A* 2021, 9, 1713-1719
6	Cu-W-TiO_2	300 W Xe lamp (350-760 nm, 200 mW cm^{-2}), 10 mg catalyst, 2.0 MPa CH_4, 0.2 MPa O_2, 30 ºC, 2 h	345	97.1	*ACS Catal.* 2022, 12, 9515-9525
7	90% Anatase TiO_2	300 W Xe lamp (300-1100 nm 130 mW cm^{-2}), 2 mg catalyst, 2.0 MPa CH_4, 0.5 MPa O_2, 25 ºC, 2 h	48.54	97.4	*J. Am. Chem. Soc.* 2022, 144, 15977-15987
8	Defective TiO_2	300 W Xe lamp, 5 mg catalyst, 1.9 MPa CH_4, 0.1 MPa O_2, 25 ºC, 2 h	22.2	73.4	*ACS Appl. Mater. Interfaces* 2022, 14, 21069-21078
9	ZnO/Fe_2O_3	300 W Xe lamp with a standard AM 1.5G filter (100 mW cm^{-2}), 0.1 MPa CH_4, 1.5 h	17.83	~100%	*J. Am. Chem. Soc.* 2022, 144, 12357-12366
10	Pd/def-In_2O_3	LED lamp (420 nm), 10 mg catalyst, 1.9 MPa CH_4, 0.1 MPa O_2, 25 ºC, 3 h	179.7	~100%	*Nat. Commun.* 2022, 13, 2930.
11	Au/WO_3	300 W Xe lamp (300-780 nm 100 mW cm^{-2}), 2 mg catalyst, 1.9 MPa CH_4, 0.1 MPa O_2, 25 ºC, 2 h	14.2	99%	*Appl. Catal. B* 2021, 283, 119661-119671.
12	Pt/WO_3	2 mg catalyst, 4 MPa CH_4, 1 MPa O_2, 30 ºC, 2 h	19.4	71	*J. Phys. Chem. Lett.* 2021, 12, 7459-7465

13	Au/BP	300 W Xe lamp (300-1100 nm, 1.2 W), 200 mg catalyst, 2.0 MPa CH_4, 0.5 MPa O_2, 90 ºC, 2 h	22.8	99	*Nat. Commun.* 2021, 12, 1218

References

[1] J. Yang, Z. Sun, K. Yan, H. Dong, H. Dong, J. Cui, X. Gong, S. Han, L. Huang, J. Wen, *Green Chem.* **2021,** *23*, 2756.

[2] J. Chen, S. Stepanovic, A. Draksharapu, M. Gruden, W. R. Browne, *Angew. Chem., Int. Ed.* **2018**, *57*, 3207. *Angew. Chem*. **2018**, *130*, 3261.

[3] J.-J. Yang, Y. Zhang, X.-Y. Xie, W.-H. Fang, G. Cui, *ACS Catal.* **2022**, *12*, 8558.

[4] D. Wang, T. Sheng, J. Chen, H.-F. Wang, P. Hu, *Nat. Catal.* **2018**, *1*, 291.

[5] G. Kresse, J. Furthmüller, *Phy. Rev.B* **1996,** *54*, 11169.

[6] J. P. Perdew, K. Burke, M. Ernzerhof, *Phy. Rev. Lett.* **1996,** *77*, 3865.

[7] G. Kresse, D. Joubert, *Phy. Rev. B* **1999,** *59*, 1758.

[8] S. Grimme, J. Antony, S. Ehrlich, H. Krieg, *J. Chem. Phys.* **2010,** *132*, 154104.

[9] G. Henkelman, H. Jónsson, *J. Chem. Phys.* **1999,** *111*, 7010.

[10] G. Henkelman, H. Jónsson, *J. Chem. Phys.* **2000,** *113*, 9978.

[11] G. Henkelman, B. P. Uberuaga, H. Jónsson, *J. Chem. Phys.* **2000,** *113*, 9901.

[12] K. Mathew, R. Sundararaman, K. Letchworth-Weaver, T.A. Arias, R.G. Hennig, *J. Chem. Phys.* **2014**, 140, 084106.

[13] J. Schneider, D. W. Bahnemann *J. Phys. Chem. Lett.* **2013**, *4*, 3479.
